# GF-Predictability for Dental Implants (GF-PreDImp): A Multidomain Predictive Model for Dental Implant Success—Development, Structure and Clinical Application (Project Report)

**DOI:** 10.3390/bioengineering13050590

**Published:** 2026-05-21

**Authors:** Gustavo Vicentis Oliveira Fernandes, Juliana Campos Hasse Fernandes, Sérgio A. Gehrke

**Affiliations:** 1 Missouri School of Dentistry and Oral Health, A.T. Still University, St. Louis, MO 63104, USA; 2 GF10 Foundation, St. Louis, MO 63104, USA; 3 Department of Pharmaceutical Science, School of Health Sciences, Vale do Itajai University (UNIVALI), Itajai 88302-901, Brazil; 4 Department of Implantology, Bioface, Postgrados en Odontologia, Universidad Catolica de Murcia, Montevideo 11100, Uruguay

**Keywords:** algorithm, dental implants, predictability, project report, risk factors

## Abstract

Dental implant therapy demonstrates high long-term survival; however, biological, behavioral, and technical complications remain prevalent. The objective of this project report was to introduce GF-Predictability for Dental Implants (GF-PreDImp), a novel, comprehensive pre-surgical multidimensional scoring proposal designed to quantify implant success predictability through a structured, evidence-based system. The model integrates six domains, Biological, Behavioral, Hard tissue, Soft tissue, Implant, and Prosthetic, assessing variables into a 100-point composite index. The domains evaluate systemic conditions (20 pts), behavioral habits (20 pts), hard-tissue anatomy (20 pts), soft-tissue characteristics (15 pts), implant parameters (15 pts), and prosthetic/surgical factors (10 pts). The final GF-PreDImp score categorizes predictability into five levels: excellent (≥85), good (70–84), moderate to guarded (55–69), guarded to high risk (40–54), and poor (<40). The tool generates dynamic visual outputs, including radar charts, enabling rapid clinical interpretation. While GF-PreDImp provides a framework for individualized risk stratification, it currently serves as a design proposal. Its implementation can improve clinical decision-making and enhance long-term implant outcomes. Further clinical assessments must be done to confirm the findings in future studies.

## 1. Introduction

Dental implants have proven to be a promising and successful substitute for missing or broken teeth. A study conducted in the United States observed a large increase in the demand for dental implants from 0.7% (1999–2000) to 5.7% (2015–2016). Annually, millions of implants are placed worldwide, with 3 million placed in the USA alone, at an increasing trend of 500,000/year. According to a report by the International Market Analysis Research and Consulting Group (IMARC), in 2022 alone, about 8 lakh dental implant and abutment procedures were carried out in India, a figure that will continue to increase over time [1]. The reported long-term survival rates exceed 90% [2,3]. Despite this success, implant failure and peri-implant diseases remain significant clinical challenges, influenced by a complex interaction of systemic, local, and behavioral factors. Current evidence suggests that implant outcomes are determined not by a single variable but by the cumulative effect of multiple risk indicators [4].

Smoking, systemic diseases such as diabetes, and poor oral hygiene have consistently been identified as major contributors to implant failure. For instance, smoking has been associated with significantly increased implant loss, with failure rates reported to be more than double those of non-smokers [5,6]. Additionally, meta-analytic data indicate that smokers may present up to a twofold increased risk of early implant failure compared to non-smokers [7]. Similarly, a history of periodontitis and inadequate keratinized mucosa has been linked to peri-implant complications and early implant loss [8,9].

The long-term success of dental implants is contingent upon mitigating a complex interplay of systemic, behavioral, and local risk factors that can jeopardize osseointegration and trigger peri-implant diseases. Smoking remains one of the most critical behavioral hazards, with smokers facing a 140.2% higher risk of implant failure compared to non-smokers due to impaired blood flow, reduced oxygenation, and delayed wound healing [10,11]. Beyond tobacco use, systemic conditions such as uncontrolled diabetes and a history of chronic periodontitis, which can increase the risk of peri-implantitis by 4.7- to 9-fold, serve as significant risk indicators for progressive bone loss [12,13]. Furthermore, iatrogenic and local factors, including poor oral hygiene, residual cement, and noncompliance with supportive periodontal maintenance, create a “race for the surface” in which bacterial biofilms can outpace host cell integration, ultimately leading to premature implant loss [12,13].

The integration of digital and electronic technologies has revolutionized dental implant assessment, shifting the focus from subjective clinical judgment to objective, data-driven precision. Algorithm tools [14,15], artificial intelligence (AI), and Deep Learning (DL) models, particularly those utilizing Convolutional Neural Networks (CNNs), are now capable of predicting implant success with accuracies exceeding 94% by analyzing preoperative Cone-Beam Computed Tomography (CBCT) scans, outperforming even senior clinicians in identifying subtle radiographic risk markers [16,17]. Furthermore, electronic tools such as AI-driven insertion torque analysis and Resonance Frequency Analysis (RFA) provide real-time, quantifiable data on primary stability, allowing for a validated assessment of immediate loading feasibility with sensitivities as high as 90.5% [18]. These digital workflows, which include dynamic navigation systems and robotic-assisted placement, minimize iatrogenic risks by ensuring sub-millimeter accuracy in implant positioning, thereby significantly reducing the potential for neurovascular injury or biomechanical failure [19].

In addition, scientific research has led to the development of biocompatible and multifunctional biomaterials with unique and specific chemical, mechanical, and biological properties. Contemporary dental implant therapy increasingly integrates biologically active materials to optimize clinical outcomes. The use of bovine pericardium membranes specifically offers several advantages: they are biocompatible, possess suitable mechanical properties, and promote wound healing due to their natural collagen structure. Clinical evidence demonstrates that these membranes can improve both the quantity and quality of regenerated tissues, reducing peri-implant complications [20].

Despite the growing body of evidence, clinicians often lack a unified, scientifically based system that integrates both pre-surgical and biomechanical predictors into a single framework. Existing systems often focus strictly on peri-implant disease risk post-placement. The rationale for developing a pre-surgical tool is to allow clinicians to visually map and mitigate interdisciplinary risks before surgical intervention, potentially reducing the incidence of early failure and long-term complications. Therefore, the aim of this study was to introduce GF-Predictability for Dental Implants (GF-PreDImp), a multidimensional scoring proposal designed to quantify implant predictability and support evidence-based clinical decision-making.

## 2. Materials and Methods

### 2.1. Conceptual Development of GF-PreDImp

The GF-PreDImp model, an algorithmic system, was developed on the premise that implant success is multifactorial and requires the simultaneous evaluation of systemic, behavioral, anatomical, and prosthetic variables. The design of the tool was guided by three principles: integration of multiple domains, weighting based on clinical relevance, and real-time usability, resulting in six domains: Biological, Behavioral, Hard tissue, Soft tissue, Implant, and Prosthetic.

Predictors included in the model were selected based on their statistical significance and clinical relevance as reported in the literature. Studies evaluating implant failure consistently identify smoking, bone quality, implant characteristics, and periodontal history as key determinants of outcome [21]. Furthermore, peri-implant disease has been shown to be associated with patient-related factors, including systemic health and oral hygiene, reinforcing the need for a comprehensive assessment model [8].

The resulting framework is a 100-point composite scoring system that allows clinicians to classify implant predictability into five categories: Excellent, Good, Moderate/Guarded, Guarded/High Risk, and Poor.

### 2.2. Structure of the GF-PreDImp

The GF-PreDImp tool is structured into six domains, each representing a critical dimension of implant success. These domains are weighted according to their relative contribution to clinical outcomes.

The biological/systemic domain evaluates systemic conditions that influence healing and osseointegration. Conditions such as uncontrolled diabetes, osteoporosis, cardiovascular disease, and immunosuppression are known to impair bone metabolism and tissue repair, thereby increasing the risk of implant failure.

The behavioral/external domain incorporates modifiable patient-related factors. Smoking remains one of the most extensively documented risk factors, with evidence demonstrating increased implant failure rates, greater marginal bone loss, and a higher incidence of peri-implantitis among smokers [5,6]. Poor oral hygiene further exacerbates these risks, contributing to peri-implant inflammation and disease progression.

The hard tissue domain evaluates bone-related parameters, including bone quality, volume, and anatomical location. Adequate bone density and quantity are essential for achieving primary stability and successful osseointegration. Studies have shown that bone condition and implant location significantly influence failure rates, particularly in compromised sites [21].

The soft tissue domain focuses on peri-implant mucosal characteristics. The width of keratinized mucosa has been identified as a strong predictor of implant success, with insufficient keratinized tissue significantly increasing the risk of early failure [9]. Additionally, periodontal history and inflammatory parameters such as bleeding on probing play a critical role in long-term peri-implant stability.

The implant parameter domain assesses factors related to implant design and placement, including implant dimensions, surface characteristics, primary stability, and loading protocols. Evidence indicates that implant length, diameter, and design are associated with failure risk, particularly in compromised bone conditions [21].

Finally, the prosthetic/surgical domain evaluates biomechanical and operator-related factors [22]. Occlusal overload, bruxism, and prosthetic design variables such as cantilever length can significantly affect implant longevity by increasing mechanical stress and contributing to bone loss [23].

### 2.3. GF-PreDImp Score

Six scored domains are present, totaling a 100-point GF-PreDImp Score:

(1) Biological/Systemic (20 pts)—Diabetes (HbA1c), bisphosphonates, H&N radiation, CVD, osteoporosis, immunosuppression;
(2) Behavioral/External (20 pts)—post-implant smoking, oral hygiene at follow-up, plaque/calculus index, brushing, alcohol, compliance;
(3) Hard Tissue (20 pts)—Bone quality D1–D4, bone quantity (QCT-based), jaw/arch position, GBR, sinus lift, CBCT height/width;
(4) Soft Tissue (15 pts)—Keratinized mucosa width (key predictor), periodontal history, gingival biotype, BoP, probing depth;
(5) Implant Parameters (15 pts)—Tooth position, loading timing, ISQ/primary stability, length/diameter, surface treatment; and
(6) Prosthetic/Surgical (10 pts)—Bruxism, occlusal contacts, crown-to-implant ratio, cantilever, surgeon experience, antibiotic protocol.

The final GF-PreDImp score could be excellent (≥85), good (70–84), moderate to guarded (55–69), guarded to high risk (40–54), and poor (<40) (Table 1). The formula for calculation is: GF-PreDImp Score = 100−(Total Penalty Points). The total penalty is the sum of all 6 domain penalties, which is displayed in a spider chart.

## 3. Results

### 3.1. Visualization and Functional Interface

A distinctive feature of GF-PreDImp is its integration of real-time visual analytics. The system translates numerical scores into intuitive graphical outputs, including a semicircular gauge representing overall predictability, a six-axis radar chart illustrating domain distribution, and color-coded domain bars indicating performance in each category.

**Figure 1:**
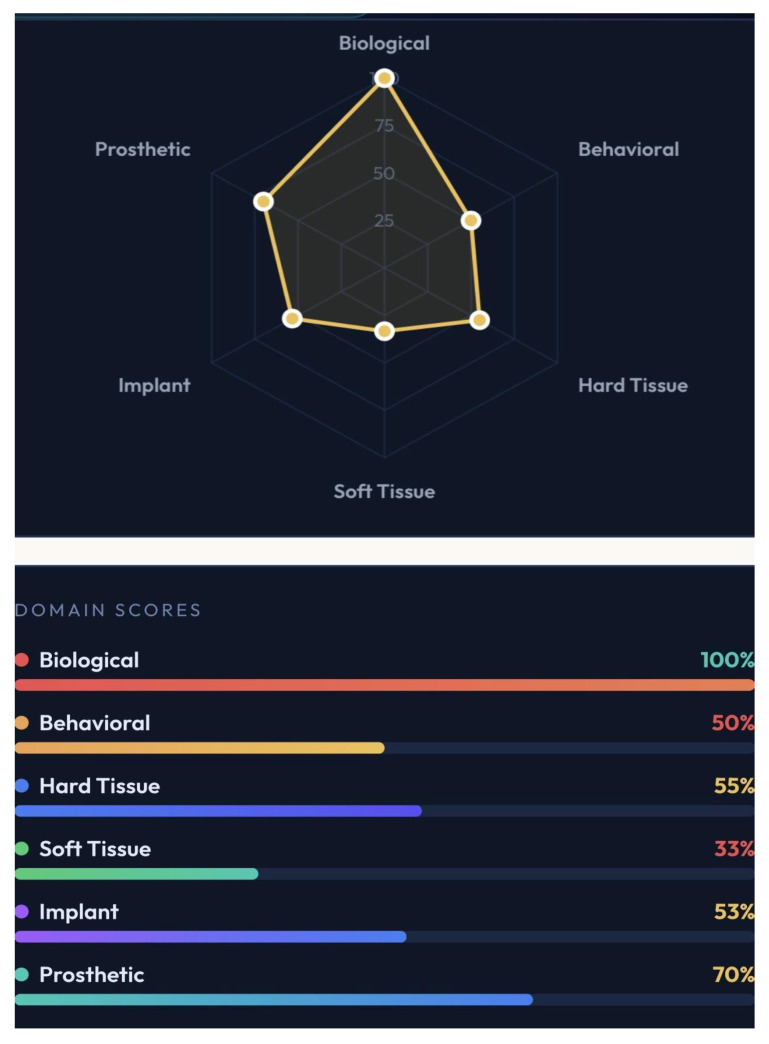
A case was developed for pre-surgery assessment using GF-PreDImp, presenting the spider chart as the result (moderate/guarded predictability) and the percentage achieved by each domain.

**Figure 2:**
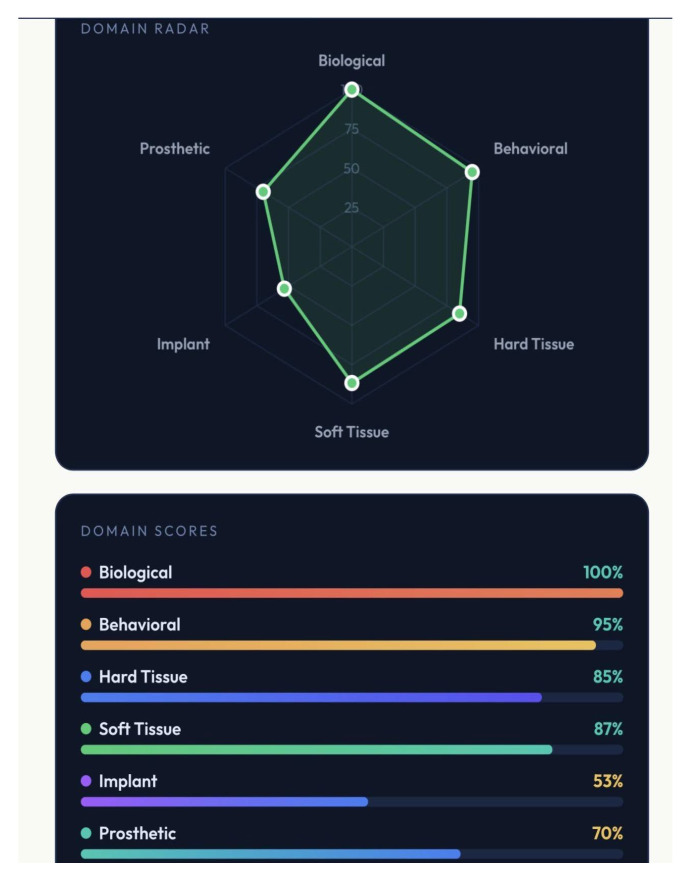
Another case, pre-surgical assessment using GF-PreDImp, showing the spider chart (good predictability) and the percentage achieved across the domains.

**Figure 3:**
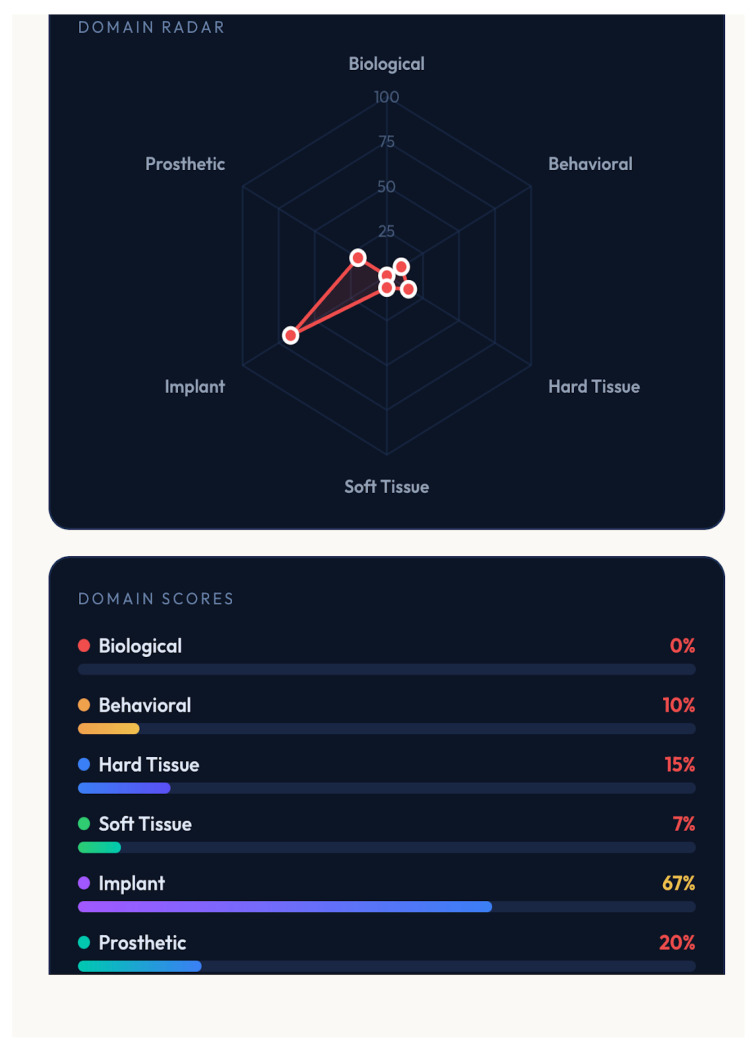
Another case, pre-surgical assessment using GF-PreDImp, showing the spider chart (poor predictability) and the percentage achieved across the domains.

The radar chart allows rapid identification of weak domains, facilitating targeted intervention. For example, the biological domain achieved the highest score, whereas the soft tissue and behavioral domains scored lower. This pattern suggests that systemic conditions are favorable, but local tissue conditions and patient habits may compromise long-term success. Figure 1, Figure 2 and Figure 3 show simulations of patients and the outcomes reached. The colors in the spider charts represent the achieved score and the verdict on predictability.

**Figure 4:**
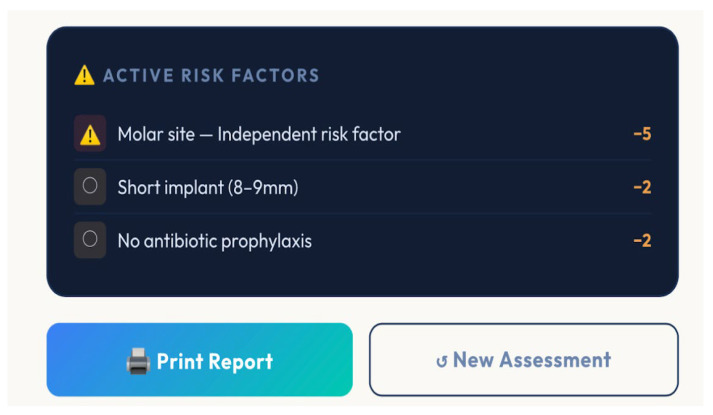
Risk factors correlated with the patient’s case.

**Figure 5:**
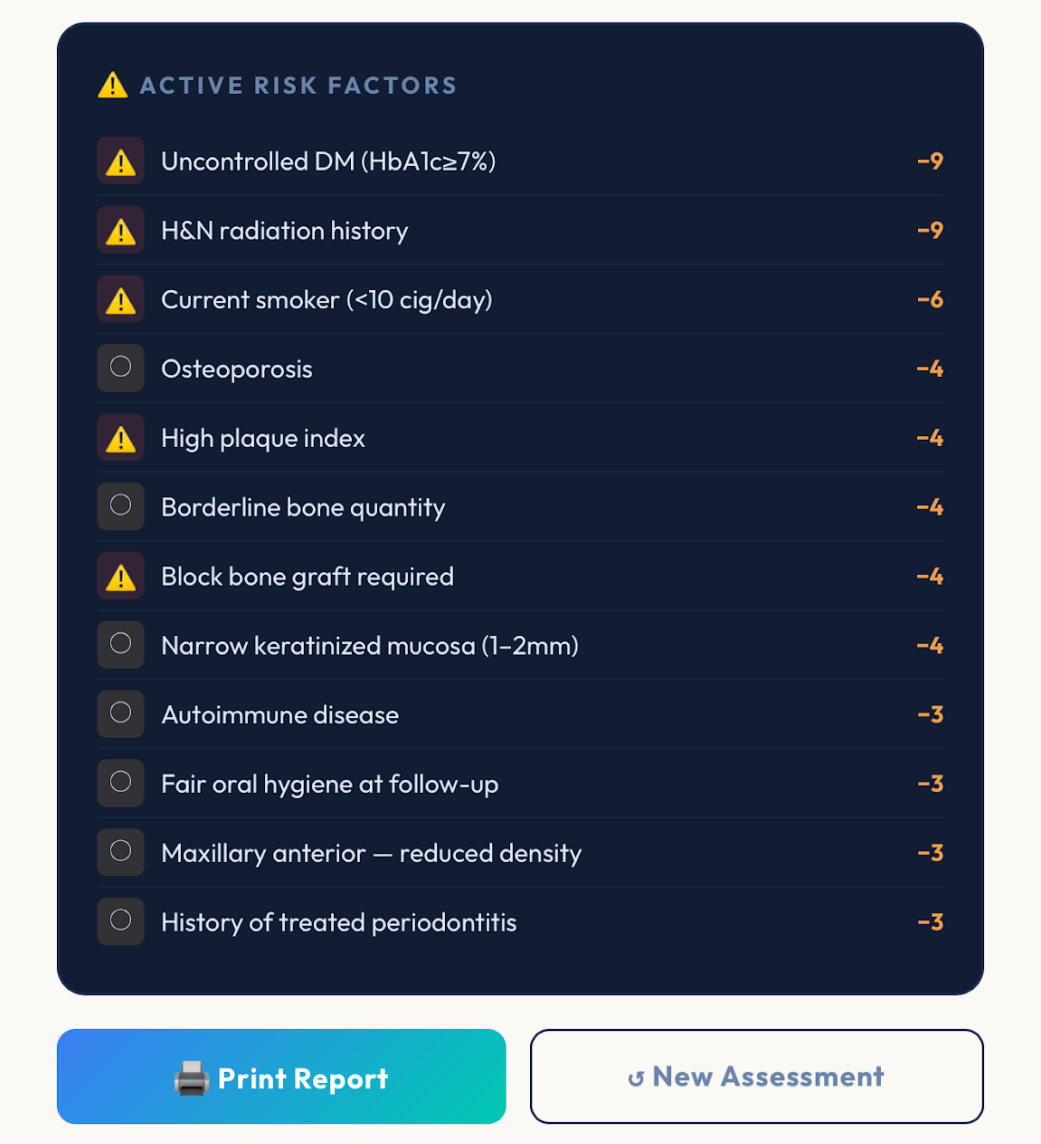
Risk factors were found that correlated with the patient’s case.

Additionally, the tool generates a ranked list of active risk factors, enabling clinicians to prioritize modifiable variables, such as smoking cessation or improved oral hygiene (Figure 4 and Figure 5).

### 3.2. Clinical Interpretation and Application

The GF-PreDImp score provides a quantitative basis for clinical decision-making. Integrating multiple risk factors into a single index allows clinicians to identify high-risk patients and implement preventive strategies.

For instance, patients with moderate scores due to behavioral factors may benefit from preoperative interventions such as smoking cessation programs and hygiene reinforcement. Evidence suggests that smoking cessation can significantly improve implant outcomes by reducing complications and enhancing healing.

Similarly, deficiencies in soft tissue parameters may indicate the need for mucogingival procedures prior to implant placement. Given the strong association between keratinized mucosa and implant success, such interventions can substantially improve predictability.

## 4. Discussion

### 4.1. The Shift Toward Holistic Risk Assessment

The GF-PreDImp model represents a crucial paradigm shift from isolated risk assessment toward a holistic, integrated approach in implant dentistry. Traditional models have historically focused on individual, siloed variables—such as isolated bone quality or specific systemic diseases—often failing to account for the synergistic interactions between these factors. However, contemporary evidence clearly demonstrates that implant outcomes, particularly long-term survival and prevention of peri-implantitis, are determined by the cumulative effects of multiple systemic, local, and behavioral variables. By generating a composite 100-point score, the GF-PreDImp system parallels and expands upon the philosophies of established tools such as the Periodontal Risk Assessment (PRA) [24] and the Implant Disease Risk Assessment (IDRA) [25], while introducing a broader, pre-surgical predictive scope that encompasses both prosthetic and anatomical parameters.

### 4.2. Biological and Behavioral Interplay

The inclusion of behavioral and systemic variables as heavily weighted domains (20 points each) is particularly relevant, as these factors directly dictate the physiological environment for osseointegration. Poor glycemic control in diabetic patients (as measured by HbA1c) and the use of bone-modifying agents significantly impair bone metabolism and wound healing [26]. Furthermore, behavioral factors are uniquely modifiable. Smoking, for example, exerts a deleterious effect on both systemic and local environments by impairing angiogenesis, altering the oral microbiome, and increasing the local inflammatory response. This drastically increases the risk of early implant failure and late peri-implant disease [5,7]. By quantifying these risks, the GF-PreDImp model flags the immediate need for pre-surgical interventions, such as smoking cessation protocols or medical consultations for glycemic control.

### 4.3. The Critical Role of Local and Biomechanical Factors

The emphasis on soft- and hard-tissue characteristics reflects a robust body of evidence highlighting their critical role in maintaining peri-implant health. The presence of adequate keratinized mucosa is no longer viewed merely as an aesthetic requirement but as a biological imperative that facilitates proper plaque control, improves mucosal seal, and reduces the incidence of mucosal recession and marginal bone loss [27].

Similarly, the inclusion of the prosthetic/surgical domain addresses the mechanical realities of implant loading. Biomechanical overload is heavily influenced by the patient’s intrinsic bite force capability. The risk for overload and subsequent implant failure is minor in a patient with a bite force capability of 100 Newtons. Conversely, a patient capable of generating 2000 Newtons of force presents a substantially higher risk of inducing micromovements exceeding 100 microns. Such micromotion disrupts initial healing, favoring fibrous encapsulation over osseointegration. Integrating these biomechanical forces into the predictive algorithm ensures that the surgical plan is structurally aligned to withstand the patient’s specific functional demands. Thus, variables such as occlusal overload, untreated bruxism, and unfavorable crown-to-implant ratios are well-documented catalysts for mechanical complications (e.g., screw loosening, fracture) and progressive peri-implant bone loss [28]. Integrating these biomechanical factors into the predictive algorithm ensures that the surgical plan is fundamentally aligned with the final prosthetic reality.

### 4.4. Visual Analytics in Shared Decision-Making

A defining strength of the GF-PreDImp tool is its real-time visual analytics, particularly the generation of intuitive spider charts. In modern clinical practice, effective communication of risk is as important as the clinical execution of the surgery itself. Translating complex, multi-dimensional clinical data into a color-coded visual format enhances patient health literacy. It empowers patients to visually comprehend how their personal habits (e.g., poor oral hygiene, smoking) directly distort their “predictability web.” This fostered shared decision-making, improved informed consent, and strongly incentivized patient compliance with preoperative instructions and postoperative maintenance [29].

### 4.5. Limitations and Future Directions

Despite its potential, the GF-PreDImp model has fundamental limitations inherent to a project report and design proposal. First, the tool lacks current clinical validation. In its present form, it is a methodological proposal derived from narrative literature rather than a validated instrument. Prospective clinical studies and randomized trials are required to correlate preoperative GF-PreDImp scores with actual 5- and 10-year implant survival metrics.

Second, the weighting system is currently unvalidated. The point allocation across domains (20, 20, 20, 15, 15, 10) is based on the author’s review of the literature rather than formal methodology. To mitigate potential operator bias, future iterations must employ a consensus-based decision-making framework, such as a formal Delphi process involving multiple expert groups, and multivariate regression models to calibrate the point penalties accurately. Finally, some variables rely on subjective clinical judgment (e.g., surgeon experience), which may introduce inter-operator variability. As the dataset grows, integrating Machine Learning (ML) algorithms will be crucial to dynamically adjust penalty weights based on real-world longitudinal outcomes.

## 5. Conclusions

GF-PreDImp has been published for the first time in the literature, showing a comprehensive and clinically applicable framework for assessing dental implant predictability. Integrating six critical domains into a unified scoring system enables clinicians to quantify risk, identify modifiable factors, and optimize treatment strategies.

The tool represents an important step toward personalized implant dentistry, transforming complex clinical data into actionable insights. Future validation and integration with digital and artificial intelligence platforms may further enhance its predictive accuracy and clinical utility.

## Figures and Tables

**Table 1:** Score interpretation.

Score Range	Verdict	Clinical Meaning
≥85	Excellent Predictability	5-year survival > 95%—Proceed with confidence
70–84	Good Predictability	High success likelihood—Manage identified risks
55–69	Moderate/Guarded	Guarded prognosis—Risk modification required
40–54	Guarded/High Risk	High risk—Address contraindications before proceeding
<40	Poor Predictability	Multiple major risk factors—Reconsider implant therapy

## Data Availability

Requests can be made directly to the corresponding author.
